# Supplementary material for: Assessment of three antibiotic combination regimens against Gram-negative bacteria causing neonatal sepsis in low- and middle-income countries
Source: Nat Commun. 2024 May 10;15:3947. doi: 10.1038/s41467-024-48296-z (PMC11087563; doi:10.1038/s41467-024-48296-z)
Supplement: Supplementary file 1 — Supplementary Information [file 41467_2024_48296_MOESM1_ESM.pdf]

Supplementary Table 1. In vitro susceptibility of *K. pneumoniae* (n=87) *and E. coli* (n=21) isolates to selected antimicrobial agents.

**Antibiotic**	**MIC ranges**	**MIC_{50} (mg/L)**	**MIC_{90} (mg/L)**	**EUCAST R%**
K. pneumoniae				
Ampicillin	8- >128	>128	>128	98
Piperacillin-Tazobactam	8/4- >256/4	32/4	>256/4	65
Flomoxef	0.06- >128	0.25	>128	34*
Cefotaxime	0.03- >128	>128	>128	85
Meropenem	0.03- >128	0.06	>128	30
Amikacin	1- >256	4	>256	31
Gentamicin	0.25- >256	64	>256	74
Fosfomycin	1- >512	8	64	11
E. coli				
Ampicillin	4->128	>128	>128	90
Piperacillin-Tazobactam	1/4->256/4	2/4	32/4	33
Flomoxef	<=0.06- >128	0.12	0.5	10*
Cefotaxime	0.06->128	0,5	>128	47
Meropenem	0.03-32	0.03	0.06	5
Amikacin	2->256	4	32	14
Gentamicin	0.5->256	2	256	38
Fosfomycin	0.5->512	0.5	1	5

*Isolates were reported as susceptible to flomoxef up to a MIC of ≤1 mg/L [34]

MIC ranges, MIC_{50}, MIC_{90} values and percentages of resistance are according to the EUCAST clinical breakpoints (v13.0, January 2023)

Supplementary Fig. 1

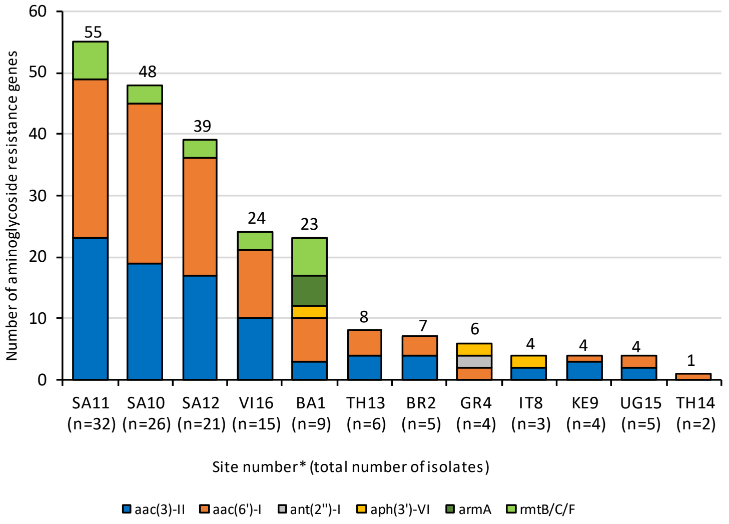

Supplementary Fig. 1. Distribution of aminoglycoside resistance genes of K. pneumoniae isolates (n=135) by site. (n) = total number of K. pneumoniae isolates per site found. Two sites that collected K. pneumoniae isolates (BR3 n=3 and TH14 n=2) without aminoglycoside resistance genes being detected, are not represented in this figure. Several isolates were carrying more than one resistance gene to a given class of antimicrobial agents. Other aminoglycoside resistance genes: aadA, aph(6')-I (streptomycin resistance) and aph(3')-I (neomycin/kanamycin resistance) were very frequent but are not represented in this figure due to their lack of relevance for the treatment of sepsis in humans. *VI: Vietnam, UG: Uganda, TH: Thailand, SA: South Africa, KE: Kenya, IT: Italy, GR: Greece, BR: Brazil, BA: Bangladesh. The numbers following the country keys refer to the site number. Source data are provided as a Source Data file.

Supplementary Fig. 2

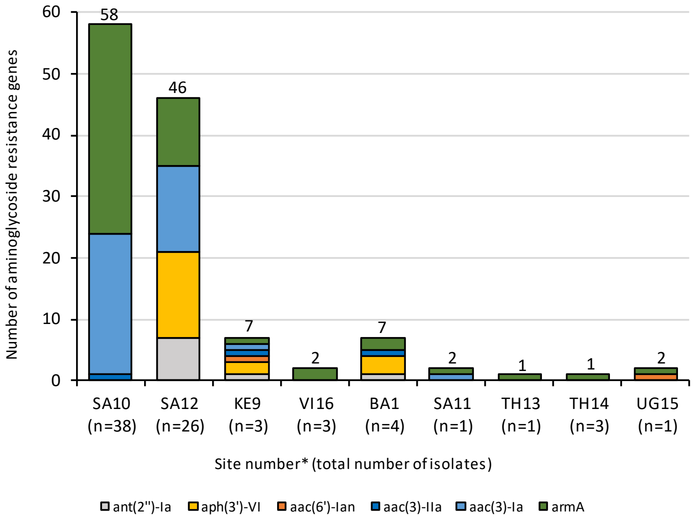

Supplementary Fig. 2. Distribution of aminoglycoside resistance genes of *A. baumannii* isolates (n=80) per site. (n) = total number of *A. baumannii* isolates collected per site. *aph(3')-VI* (n=2) and *aph(3')-VIa* (n=17) are grouped together. Several isolates carried more than one resistance gene to a given class of antimicrobial agents. *VI: Vietnam, UG: Uganda, TH: Thailand, SA: South Africa, KE: Kenya, IT: Italy, GR: Greece, BR: Brazil, BA: Bangladesh. The numbers following the country keys refer to the site number. Source data are provided as a Source Data file.
