# Supplementary material for: Assessment of three antibiotic combination regimens against Gram-negative bacteria causing neonatal sepsis in low- and middle-income countries
Source: Nat Commun. 2024 May 10;15:3947. doi: 10.1038/s41467-024-48296-z (PMC11087563; doi:10.1038/s41467-024-48296-z)
Supplement: Supplementary file 3 — Description of Additional Supplementary Files [file 41467_2024_48296_MOESM3_ESM.pdf]

Description of Additional Supplementary files

Supplementary Data 1. Minimal inhibitory concentrations (MICs) for all *E. coli* and *K. pneumoniae* strains in NeoOBS study tested against six antibiotics and their susceptibility against the three new antibiotic combinations (fosfomycin/flomoxef; fosfomycin/amikacin and flomoxef/amikacin). AMP: ampicillin; GEN: gentamycin; TZP: piperacillin-tazobactam; CTX: cefotaxime; MEM: meropenem; FLX: flomoxef; AMK: amikacin; FOS: fosfomycin.

Supplementary Data 2. List of Accession IDs of sequencing data generated in the study.
