# Supplementary material for: Assessment of three antibiotic combination regimens against Gram-negative bacteria causing neonatal sepsis in low- and middle-income countries
Source: Nat Commun. 2024 May 10;15:3947. doi: 10.1038/s41467-024-48296-z (PMC11087563; doi:10.1038/s41467-024-48296-z)
Supplement: Supplementary file 6 — Reporting Summary [file 41467_2024_48296_MOESM6_ESM.pdf]

nature portfolio

Corresponding author(s): Biljana Kakaraskoska Boceska; Youri Glupczynski

Last updated by author(s): Apr 4, 2024

Reporting Summary

Nature Portfolio wishes to improve the reproducibility of the work that we publish. This form provides structure for consistency and transparency in reporting. For further information on Nature Portfolio policies, see our Editorial Policies and the Editorial Policy Checklist.

Statistics

For all statistical analyses, confirm that the following items are present in the figure legend, table legend, main text, or Methods section.

n/a	Confirmed	
☐	☒	The exact sample size (*n*) for each experimental group/condition, given as a discrete number and unit of measurement
☐	☒	A statement on whether measurements were taken from distinct samples or whether the same sample was measured repeatedly
☐	☒	The statistical test(s) used AND whether they are one- or two-sided<br>*Only common tests should be described solely by name; describe more complex techniques in the Methods section.*
☒	☐	A description of all covariates tested
☒	☐	A description of any assumptions or corrections, such as tests of normality and adjustment for multiple comparisons
☒	☐	A full description of the statistical parameters including central tendency (e.g. means) or other basic estimates (e.g. regression coefficient) AND variation (e.g. standard deviation) or associated estimates of uncertainty (e.g. confidence intervals)
☐	☒	For null hypothesis testing, the test statistic (e.g. *F*, *t*, *r*) with confidence intervals, effect sizes, degrees of freedom and *P* value noted<br>*Give P values as exact values whenever suitable.*
☒	☐	For Bayesian analysis, information on the choice of priors and Markov chain Monte Carlo settings
☒	☐	For hierarchical and complex designs, identification of the appropriate level for tests and full reporting of outcomes
☒	☐	Estimates of effect sizes (e.g. Cohen's *d*, Pearson's *r*), indicating how they were calculated

Our web collection on statistics for biologists contains articles on many of the points above.

Software and code

Policy information about availability of computer code

Data collection	Samples were collected using REDCap™ 10.0.12. during the clinical trial NCT03721302 and were cultured at local sites. Isolates were sent to UAntwerpen. Isolate data were extracted from the eCRFs.
Data analysis	WGS data were analyzed by:<br>- Quality check and trimming of the raw data of the sequenced strains done with trimmomatic v.0.4.2<br>- The secondary analysis performed using BacPipe v.1.2.6 (https://github.com/wholeGenomeSequencingAnalysisPipeline/BacPipe)<br>- de novo assembly by SPAdes (v.3.11.0) and sequence annotation with Prokka (v1.11.1)<br>- quality check of assembled contigs by checkM (V1.1.6)<br>- Resistomes of the strains were determined using the ResFinder v.2.1 and CARD v.5.1 databases<br>- For core genome multilocus sequence typing (cgMLST), a gene-by-gene approach using ChewBBACA (V.3.1.2)<br>- Trees were visualized using Grapetree (V.1.5.0) (https://github.com/achtman-lab/GrapeTree/releases)<br>- For MLST analysis was used PubMLST.org

For manuscripts utilizing custom algorithms or software that are central to the research but not yet described in published literature, software must be made available to editors and reviewers. We strongly encourage code deposition in a community repository (e.g. GitHub). See the Nature Portfolio guidelines for submitting code & software for further information.

Data

Policy information about availability of data

All manuscripts must include a data availability statement. This statement should provide the following information, where applicable:

- Accession codes, unique identifiers, or web links for publicly available datasets
- A description of any restrictions on data availability
- For clinical datasets or third party data, please ensure that the statement adheres to our policy

The raw sequencing data (FASTQ) generated in this study have been deposited in NCBI database under BioProject number: PRJNA1087366

Research involving human participants, their data, or biological material

Policy information about studies with human participants or human data. See also policy information about sex, gender (identity/presentation), and sexual orientation and race, ethnicity and racism.

Reporting on sex and gender	Sex and gender characteristics were not taken in consideration for this manuscript.
Reporting on race, ethnicity, or other socially relevant groupings	There were no groupings based on race, ethnicity, or other socially relevant characteristics for this manuscript.
Population characteristics	The data presented in this paper are about microbiological characterization of bacterial strains obtained from hospitalized infants <60 days with clinical sepsis that were enrolled in the main study (NCT03721302 also at https://journals.plos.org/plosmedicine/article?id=10.1371/journal.pmed.1004179).
Recruitment	Infants <60 days with clinical sepsis were eligible if the local physician had decided to treat the infant with antibiotics for a new episode of sepsis meeting the inclusion criteria, derived by combining clinical and laboratory criteria from WHO pSBI (http://apps.who.int/iris/bitstream/handle/10665/181426/9789241509268_eng.pdf?sequence=1.) and EMA Criteria for neonatal sepsis trials (https://pubmed.ncbi.nlm.nih.gov/31170237/). A minimum of 2 clinical, or 1 clinical and 1 laboratory sepsis criteria, were required for inclusion, and up to 200 infants per site were enrolled according to a sampling frame adapted to local case volume and activity. Infants were excluded if an alternative primary diagnosis other than sepsis was suspected, or a serious non-infective co-morbidity was expected to cause death within 72 h. Previous antibiotic use was not an exclusion criterion as long as a new antibiotic regimen was being started after a blood culture for a distinct new episode of sepsis. Sepsis episodes occurring >48 h after admission, defined by time of blood culture, were defined as health-care-associated infections (HAIs). (https://journals.plos.org/plosmedicine/article?id=10.1371/journal.pmed.1004179
Ethics oversight	St. George's, University of London (SGUL) Research Ethics Committee and sites' local, central or national ethics committees and other relevant local bodies, where required.

Note that full information on the approval of the study protocol must also be provided in the manuscript.

Field-specific reporting

Please select the one below that is the best fit for your research. If you are not sure, read the appropriate sections before making your selection.

☒ Life sciences ☐ Behavioural & social sciences ☐ Ecological, evolutionary & environmental sciences

For a reference copy of the document with all sections, see nature.com/documents/nr-reporting-summary-flat.pdf

Life sciences study design

All studies must disclose on these points even when the disclosure is negative.

Sample size	For this manuscript no sample size calculation was performed as we were dependent on the number of isolates referred to us and for which identification could be confirmed to species level by MALDI-TOF MS and WGS subsequently to regrowth at culture.<br>We estimated a number of at least 20 isolates per species to be included in the microbiological analysis. This number was chosen arbitrarily but was however assumed to reflect sufficient occurrence and distribution among the different sites to assess the genetic diversity and the resistance mechanisms (ESBLs, carbapenemase, aminoglycoside resistance) of major clinical and public health importance. Results for 309 Gram-negative isolates were included containing the patient's first clinical isolate from blood and/or CSF. Isolates belonging to a given species for which less than 20 isolates were obtained were not included. To avoid analyzing replicates of the same bacterial clone, only isolates displaying different genetic profiles were selected for in vitro susceptibility testing.<br>As for the clinical study, sample size determination (of the number of neonates to be enrolled) was calculated to ensure a population which represents the diversity of neonatal sepsis management. Within each site, recruiting 200 infants with significant sepsis provides >80% power to detect differences in the estimated mortalities of 50% for blood culture positive cases vs. 10% for blood culture negative cases, a conservative estimate of blood culture positivity rate (per admission to NICU) is around 5% and observing 50% mortality in blood-culture positive vs. 10% in blood culture negative.
Data exclusions	Data from 2 countries, which where originally included in the study, were not reported in our manuscript. Isolates from these countries were

Data exclusions	not provided in our laboratory. Isolates belonging to a given species for which less than 20 isolates were obtained were not included.
Replication	To guarantee the precision and consistency of WGS sequencing data, we incorporated intra sequencing run positive controls and sequencing library preparation positive and negative controls into every sequencing library preparation and DNA extraction process. This process was validated every five experiments and it was successful in every attempt. Subsequently, the raw sequencing data and assemblies underwent thorough quality and cross contamination checks after the sequencing run (data not displayed).
Randomization	N/A. This is a microbiological study that focuses on the epidemiology of antimicrobial resistance in Gram-negative bacteria. We performed the different analysis on the bacterial clinical isolates that had been collected at different local sites and were made available to use.<br>We selected the strains belonging to the most common GNB species in order to characterize these phenotypically and genotypically for resistance mechanisms and molecular epidemiological typing by MLST. No direct link was established between microbiological characters of the strains with clinical outcome in this study and investigators were anyhow not aware of the origin of the strains (coded numbers) at the time they were performing the analysis.
Blinding	N/A. Please see above, same as for randomization.

Reporting for specific materials, systems and methods

We require information from authors about some types of materials, experimental systems and methods used in many studies. Here, indicate whether each material, system or method listed is relevant to your study. If you are not sure if a list item applies to your research, read the appropriate section before selecting a response.

Materials & experimental systems

n/a	Involved in the study
☒	☐ Antibodies
☒	☐ Eukaryotic cell lines
☒	☐ Palaeontology and archaeology
☒	☐ Animals and other organisms
☒	☐ Clinical data
☒	☐ Dual use research of concern
☒	☐ Plants

Methods

n/a	Involved in the study
☒	☐ ChIP-seq
☒	☐ Flow cytometry
☒	☐ MRI-based neuroimaging

Plants

Seed stocks	N/A
Novel plant genotypes	N/A
Authentication	N/A
